# Efficiency limits of concentrating spectral-splitting hybrid photovoltaic-thermal (PV-T) solar collectors and systems

**DOI:** 10.1038/s41377-021-00465-1

**Published:** 2021-02-05

**Authors:** Gan Huang, Kai Wang, Christos N. Markides

**Affiliations:** 1 Clean Energy Processes (CEP) Laboratory, Department of Chemical Engineering, Imperial College London, London, SW7 2AZ UK; 2 Institute of Refrigeration and Cryogenics, Key Laboratory of Refrigeration and Cryogenic Technology of Zhejiang Province, Zhejiang University, Hangzhou, 310027 China

**Keywords:** Optical physics, Solar energy and photovoltaic technology

## Abstract

Spectral splitting is an approach to the design of hybrid photovoltaic-thermal (PVT) collectors that promises significant performance benefits. However, the ultimate efficiency limits, optimal PV cell materials and optical filters of spectral-splitting PVT (SSPVT) collectors remain unclear, with a lack of consensus in the literature. We develop an idealized model of SSPVT collectors and use this to determine their electrical and thermal efficiency limits, and to uncover how these limits can be approached through the selection of optimal PV cell materials and spectral-splitting filters. Assuming that thermal losses can be minimized, the efficiency limit, optimal PV material and optimal filter all depend strongly on a coefficient *w*, which quantifies the value of the delivered thermal energy relative to that of the generated electricity. The total (electrical plus thermal) efficiency limit of SSPVT collectors increases at higher *w* and at higher optical concentrations. The optimal spectral-splitting filter is defined by sharp lower- and upper-bound energies; the former always coincides with the bandgap of the cell, whereas the latter decreases at higher *w*. The total effective efficiency limit of SSPVT collectors is over 20% higher than those of either standalone PV modules or standalone ST collectors when *w* is in the range from 0.35 to 0.50 and up to 30% higher at *w* ≈ 0.4. This study provides a method for identifying the efficiency limits of ideal SSPVT collectors and reports these limits, along with guidance for selecting optimal PV materials and spectral-splitting filters under different conditions and in different applications.

## Optimizing the performance of hybrid photovoltaic-thermal collectors

Scientists have developed a method for predicting and optimizing the performance of hybrid photovoltaic-thermal (PVT) collectors. Photovoltaic (PV) cells use only a portion of the solar spectrum to generate electricity, limiting the efficiency of single-junction cells to 20–30%. PVT collectors improve on this overall solar utilization by recovering unused thermal energy and delivering a useful thermal output. Spectral splitting is a promising approach for improving the performance of PVT collectors by exploiting the spectral sensitivity of PV cells. Christos Markides and Gan Huang from Imperial College London, in collaboration with Kai Wang from Zhejiang University, have developed a comprehensive framework for modelling spectral-splitting PVT collectors, which they have used to determine the efficiency limits of such collectors and how these limits can be approached through the selection of optimal PV cell materials and spectral-splitting optical filters.

## Introduction

Solar energy is a clean and abundant energy source. Current methods of harvesting solar energy include solar thermal (ST) and photovoltaic (PV) technologies. The latter have attracted considerable interest in recent decades, as PV cells are able to convert solar energy directly into valuable electricity without noise and moving parts in simple systems that are easy to install^1–3^. Global PV capacity crossed the milestone of 500 GW in 2018, overtaking solar water-heating collector capacity for the first time and continuing to grow, reaching 630 GW in 2019^4^. Common solar cell materials include semiconductors, such as Si^5^, CdTe^6^, GaAs^7^, CIGS^8^ and perovskites^9^. Single-junction Si cells remain dominant within the global PV market owing to their low costs and mature manufacturing processes^10^.

Only an incident photon with a higher energy level than the bandgap of solar cells can activate an electron-hole pair and generate electricity. Thus, solar cells are sensitive to only a part of the solar spectrum that can be converted to electricity. The theoretical efficiency limit of Si solar cells is ~30% under one sun according to the seminal research of Shockley and Quisser^11^. Other solar cells also experience this partial spectral sensitivity^12^. The unused portion of the solar spectrum dissipates as waste heat in solar cells, increasing their operating temperature and leading to a monotonic deterioration in their electrical efficiency. The electrical efficiency of mono- and polycrystalline Si cells typically decreases by 4.0–6.5% for every 10 °C increase in operating temperature^13^. Radiative cooling approaches can be used to further improve these efficiencies^14^.

PVT technology has been attracting increased interest as a solution that promotes broader solar utilization. Flat-plate PVT collectors are the most common design, most of which are based on attaching a thermal absorber to the back of PV modules. The thermal absorber cools the PV cells but also harvests waste heat from the cells as useful thermal energy, which can be utilized downstream for domestic hot water or space heating^15,16^. This makes PVT collectors significantly more efficient overall than standalone PV modules^17,18^. However, in these arrangements, the thermal absorber is designed to be in good thermal contact with the PV cells, leading to similar PV cell and absorber operating temperatures and compromising the PV efficiency if a higher-temperature thermal output is pursued. This ‘thermal coupling’ feature of conventional PVT collectors unavoidably limits their performance and applications.

**Fig. 1:**
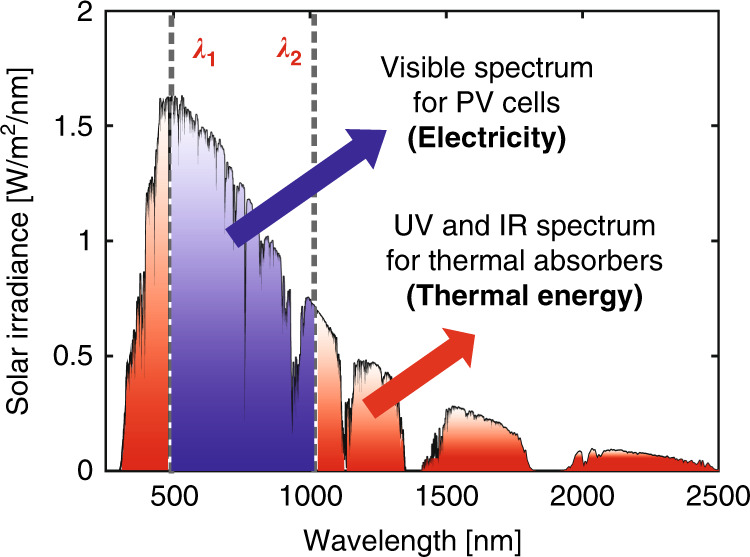
Spectral-splitting concept motivation as applied to PVT collectors.

Spectral splitting, as proposed in various forms as early as the 1970s and 1980s, is a promising approach for improving the performance of PVT collectors by exploiting the partial spectral sensitivity of PV cells while minimizing, through alternative designs, the thermal coupling in these collectors. The conceptual principle of a spectral-splitting PVT (SSPVT) collector is illustrated in Fig. 1.

As shown in this figure, in SSPVT collector concepts, the solar spectrum is separated into three different parts at wavelengths *λ*_1_ and *λ*_2_, of which only the visible part of the spectrum (*λ*_1_ < *λ* < *λ*_2_) is sent to the PV cells for electricity generation, while the rest of the spectrum in the ultraviolet (UV) and IR regions is directed to a thermal absorber. In this way, both the PV cells and thermal absorber can operate at appropriate temperatures and the SSPVT collector is able to produce very high-temperature heat without compromising the PV efficiency. High-temperature heat can be utilized in various industrial applications or, if high enough, for thermal power generation.

Current SSPVT collector concepts typically employ one of two spectral-splitting approaches based on either selectively reflective or absorptive optical filters. Selectively reflective filters, which are used widely in concentrating PVT collector designs, allow part of the solar spectrum to pass through to the PV cells and reflect the rest to a thermal absorber^19–23^. Selectively absorptive filters, on the other hand, allow only part of the solar spectrum to pass through to the PV cells and directly absorb the rest of the spectrum. Liquid flows, which can act as both optical filters and heat transfer fluids, have been proposed as promising selectively absorptive filters. Within this category, nanofluids (nanoparticle suspensions) are promising and emergent selectively absorptive filter types for SSPVT collectors, which have attracted significant interest recently^24–27^. The state-of-the-art SSPVT technology based on emerging nanomaterials has been summarized in a recent review article^28^.

The optical characteristics of the spectrum-splitting filter are crucial to the performance of SSPVT collectors, as this strongly determines the electrical, thermal and total (electrical plus thermal) efficiencies of such collectors. A number of real filters have been developed to date, including thin-film reflective and nanofluid absorptive filters. However, identifying the optimum filter, i.e., optimal values of *λ*_1_ and *λ*_2_ in Fig. 1, remains a challenge, with various studies reporting different optimum filters even for the same PV cell. The optimal wavelengths for Si cells in SSPVT collectors were reported as 751–1126 nm in Taylor et al.^25^, 732–1067 nm in Crisostomo et al.^29^, 640–1127 nm in Bierman et al.^30^, 600–1150 nm in Otanicar et al.^31^, 400–1100 nm in Shou et al.^32^, and 300–1100 nm in Soule et al.^19^. This widespread reveals a lack of consensus in the literature concerning the definition of the optimal filter, even for the most common solar cell. Furthermore, although many PV materials (Si, CdTe, GaAs and InGaP) have been applied to SSPVT collector applications, the optimal PV material for a SSPVT collector also remains unclear. Therefore, the efficiency limits of SSPVT collectors, which depend strongly on the PV material and spectral-splitting filter, remain unclear.

In this study, we develop a methodology and a model capable of rationally identifying the optimal PV cell material and spectral-splitting optical filter in different applications and of predicting the ideal efficiency limits of concentrating SSPVT collectors employing these cells and filters. The performance of SSPVT collectors in a typical application of thermal power generation is analysed. We also consider the performance of SSPVT collectors with common PV materials (Si, CIGS, CdTe, GaAs, GaInP and others) and seek to identify corresponding optimal spectral-splitting filters.

## Results

### Physical and mathematical modelling framework

**Fig. 2:**
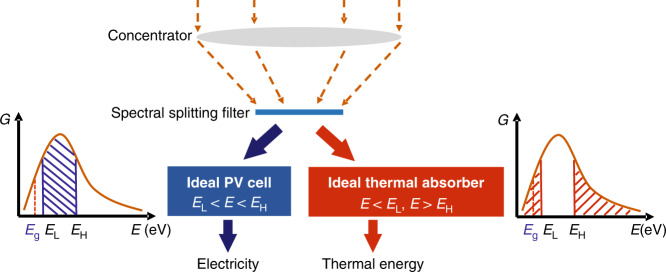
Physical model of an ideal concentrating SSPVT collector.

A physical model of an ideal concentrating SSPVT collector is shown in Fig. 2. Solar illumination is concentrated by an ideal concentrator and then spectrally separated into parts by a spectral-splitting filter according to the energy of the incident photons. Photons with energy *E*_L_ < *E* < *E*_H_ are directed to an ideal PV cell with a bandgap energy *E*_g_ and the rest (*E* < *E*_L_, *E* > *E*_H_) are absorbed by an ideal thermal absorber to generate high-temperature thermal energy. Here, *E*_L_ and *E*_H_ are defined as the lower and upper bounds of the optical filter (in eV), and the range between *E*_L_ and *E*_H_ is referred to as the ‘filter window’. Of note is that an ideal PV cell will inevitably generate waste heat even if only the desired photons are directed to the cell. The waste heat in the PV cell is challenging to recover in the form of high-temperature thermal energy both due to the low temperature limits of PV cells, which assign a low value (heat grade) to this energy, and due to the associated complexity and cost of related solutions. The SSPVT collector design employed in this study, therefore, does not attempt to recover the low-temperature waste heat in the PV cell, such that this heat is ultimately rejected to the environment.

The mathematical model in this paper is based on the following assumptions: (1) the PV cell is ideal, has a quantum efficiency of 1 and is maintained at 300 K via efficient heat rejection; (2) the optical concentrator is ideal and concentrates the incident sunlight (AM1.5) with negligible optical losses; and (3) the spectral-splitting filter ideally separates the incident photons into two parts, as shown in Fig. 2, without optical losses. The SSPVT collector model is developed on the basis of the above assumptions to determine the efficiency limits of concentrating SSPVT collectors.

The electrical efficiency of the (ideal) PV cell can be determined from:

1 $$\eta _{{\mathrm{el}}} = \frac{{{\mathrm{max}}\left( {I_{{\mathrm{PV}}} \cdot V_{{\mathrm{PV}}}} \right)}}{{\mathop {\int }\nolimits_0^\infty CG_{\mathrm{s}}(\lambda ){\mathrm{d}}\lambda }}$$

where *C* is the concentration ratio, *G*_s_(λ) is the incident solar spectral irradiance based on AM1.5, *V*_PV_ is the applied cell voltage and *I*_PV_ is the current in a single-junction solar cell^11,33,34^:

2 $$I_{{\mathrm{PV}}} = f\left( {V_{{\mathrm{PV}}},C,E_{\mathrm{L}},E_{\mathrm{H}},{\mathrm{QE}},T_{{\mathrm{PV}}},E_{\mathrm{g}}} \right)$$

where QE is the quantum efficiency, *T*_PV_ is the temperature of the PV cell and *E*_g_ is the bandgap energy of the cell. Detailed equations are introduced in the final section of this paper (i.e., ‘Materials and Methods’).

The thermal efficiency of the thermal absorber can be determined from:

3 $$\eta _{\mathrm{th}} = \frac{{E_{\mathrm{th}}}}{{\mathop {\int }\nolimits_0^\infty CG_{\mathrm{s}}\left( \lambda \right){\mathrm{d}}\lambda }} - \eta _{{\mathrm{th}},{\mathrm{loss}}}$$

where *η*_th,loss_ is the fraction of the total incident solar energy that is lost from the collector through heat transfer and *E*_th_ is the useful thermal energy gained by the thermal absorber.

Four concentration ratios (*C* = 100, 210, 1000 and 45,000) are investigated in this study, where *C* = 210 and 45,000 are taken as limits for linear concentrators (e.g., parabolic trough concentrators) and circular concentrators (e.g., parabolic dish concentrators and solar towers), respectively^35^, and *C* = 100 is taken as a typical concentration ratio that can be achieved in practice by current parabolic trough concentrators or parabolic dish concentrators^36^. Finally, *C* = 1000 is taken as a typical concentration ratio of current solar towers^36^. The heat loss ratio *η*_th,loss_ decreases as the concentration ratio increases. The heat loss can also be significantly suppressed via emissivity control and evacuation^37^. Thus, the term *η*_th,loss_ for a concentrating collector can be made small at high concentration ratios, e.g., *η*_th,loss_ of a typical evacuated tube ST collector is <1% at a concentration ratio of 100 when the output temperature is ~400 °C above the ambient temperature^37,38^. Therefore, this term is assumed to be small in this work. It is noted, however, that in cases where this assumption is not valid, this framework can be easily extended to account for realistic losses if desired, e.g., by expressing *η*_th,loss_ in terms of suitable radiative and convective loss terms.

As the values of heat and electricity are different and depend strongly on the application and the demands for these two energy vectors, a total effective efficiency can be defined as:

4 $$\eta _{{\mathrm{tot}}} = \eta _{{\mathrm{el}}} + w \cdot \eta _{{\mathrm{th}}}$$

where *w* is a weight coefficient that converts the thermal energy to an equivalent amount of electricity and reflects the worth of thermal energy relative to that of electricity. This coefficient can be based on a thermodynamic value (e.g., via second-law arguments or heat engine conversion efficiencies), a cost value (e.g., through a price ratio of heat/electricity) or a ratio of environmental benefits (e.g*.*, displaced or mitigated emissions). The optimum filter in a SSPVT collector depends on the definition of the total effective efficiency, i.e., the value of *w*. In this study, the total effective efficiency is also treated as the merit function in the optimization of the filter and the bandgap energy of the PV cell.

### Total effective efficiency limits of SSPVT collectors for different *w*

**Fig. 3:**
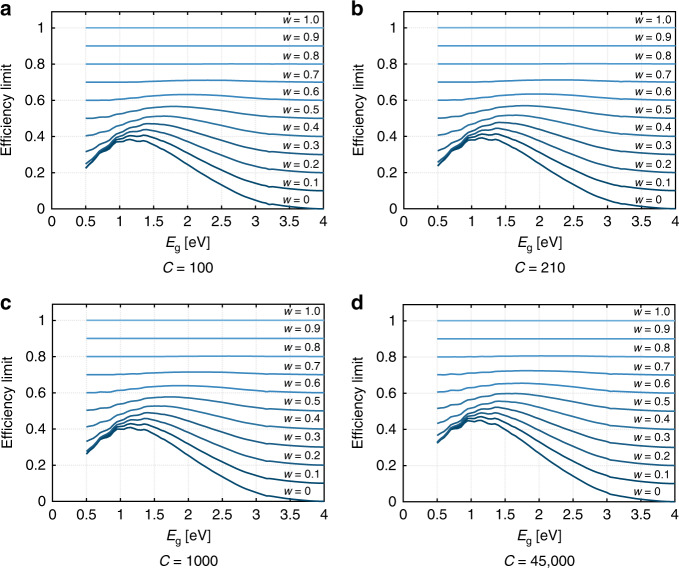
Limit of the total effective efficiency for different *w* and *C* = 100, 210, 1000, 45,000.

The total effective efficiency *η*_tot_ of SSPVT collectors reaches a maximum value for optimal values of *E*_L_ and *E*_H_. Figure 3a shows the limit of the total effective efficiency of SSPVT collectors for different PV cells with different bandgap energies and for different *w* at a concentration ratio of *C* = 100. The curve generated for *w* = 0 corresponds to the electrical efficiency limit of standalone concentrating PV cells with different bandgap energies. The thermal energy becomes more valuable (relative to electricity) as the weight coefficient *w* increases, leading to a significant increase in the limit of the total effective efficiency, as similar to pure ST collectors vis-a-vis PV modules, the conversion of sunlight to heat is more efficient than its conversion to electricity. At an extreme scenario of *w* = 1, the thermal energy has an equal value to that of the generated electricity. In this case, a ST collector delivers the highest efficiency of 100% (as we assume no heat losses at high concentrations). The total effective efficiency limit is sensitive to the solar cell bandgap energy when *w* is in the range from 0 to 0.5, corresponding to scenarios in which electricity is at least twice as valuable as thermal energy. The bandgap energy has a slight influence on the total effective efficiency limit when *w* is larger than 0.8, indicating that PV cells are less necessary when the thermal energy delivered is of comparable value to electricity. In summary, the total effective efficiency limit of SSPVT collectors depends strongly on not only the solar cell bandgap energy but also the relative value of thermal energy relative to that of electricity (i.e., *w*).

The total effective efficiency limits of SSPVT collectors with higher concentration ratios of *C* = 210, 1000 and 45,000 are shown in Fig. 3b–d. The efficiency limits shift upwards as the concentration ratio increases when the value of *w* is smaller than 0.5, but when *w* is above 0.5, this effect is minor. The standalone PV cell efficiency limit increases from 38% to 45% as *C* increases from 100 to 45,000. The limit of the total effective efficiency of SSPVT collectors thus increases as the concentration increases owing to the increased PV efficiency when *w* is below 0.5. In either case, the total effective efficiency limit is more sensitive than that of the concentration ratio, which suggests that the application is a greater determinant of performance than the solar conditions or the concentrating optical design of the system.

**Fig. 4:**
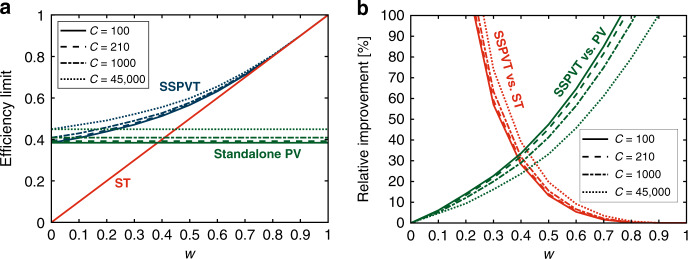
**a** Efficiency limits of SSPVT, ST and standalone PV, and **b** relative improvement of SSPVT compared to ST and PV as a function of *w* for different *C* = 100, 210, 1000, 45,000.

Also of interest are comparisons of the efficiency limits of SSPVT collectors to those of standalone single-junction PV modules and ST collectors in Fig. 4a. SSPVT collectors are able to adjust the fraction of the solar energy directed to the PV cell and to the thermal absorber according to the value of *w*, to optimize the collector and maximize the total effective efficiency. Thus, a SSPVT collector always has a higher efficiency than either standalone PV modules or standalone ST collectors when 0 < *w* < 1. The total effective efficiency limit of SSPVT collectors equals that of standalone PV modules at *w* = 0, and that of standalone ST collectors at *w* = 1. The total effective efficiency limit of SSPVT collectors also increases as the concentration ratio *C* increases.

The relative improvement offered by SSPVT collectors over ST collectors, in terms of total efficiency, decreases as *w* increases, whereas the relative improvement of SSPVT collectors over standalone PV modules increases as *w* increases, as shown in Fig. 4b. Too large or too small values of *w* decrease the advantage offered by SSPVT collectors over either standalone ST collectors or standalone PV modules. In particular, SSPVT collectors have a considerable advantage over standalone PV modules and standalone ST collectors when *w* is within a given range that depends on the concentration. For example, the total effective efficiency limit of SSPVT collectors at the maximum concentration ratio (*C* = 45,000) is over 20% higher (in relative terms) than those of both standalone PV modules and standalone ST collectors when *w* is between 0.35 and 0.50.

Of particular interest are the crossing points in Fig. 4b, which indicate design conditions for which SSPVT collectors have a maximum advantage over either standalone PV modules or ST collectors in applications where both electricity and heat are required and when all of these technologies are available for selection. To the left of these points, PV modules are preferred to ST collectors; to the right, ST collectors offer a better total efficiency than PV modules. These efficiency limit crossover points of SSPVT collectors for *C* = 100, 210, 1000 and 45,000 are 33%, 32%, 31% and 29% higher than those of standalone PV modules or ST collectors when *w* = 0.38, 0.39, 0.40 and 0.45, respectively, as shown in Fig. 4b.

**Fig. 5:**
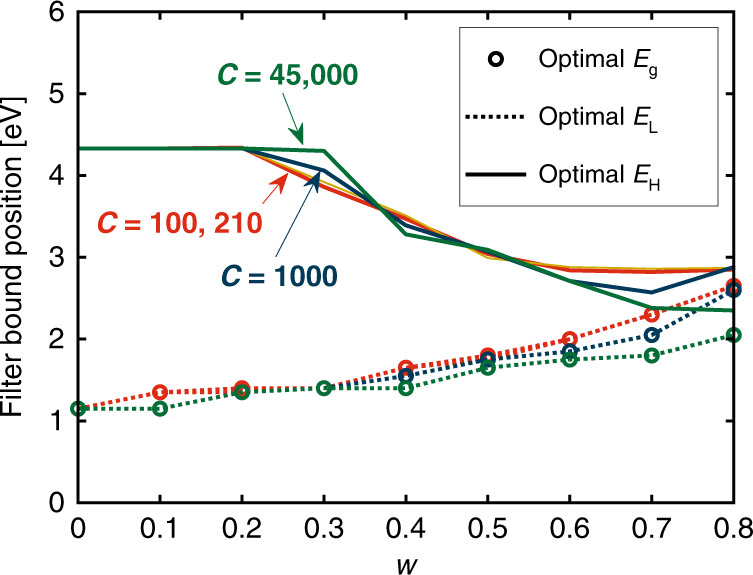
Optimal PV bandgap energy and corresponding optimal filters for different *w*. Energies (in eV) can be converted to wavelengths (in nm) via *λ*(nm) = 1240/*E*(eV), e.g., 4.4eV ≈ 280 nm.

The optimal solar cell bandgap energy *E*_g_ and the corresponding optimal lower-bound *E*_L_ and upper-bound *E*_H_ for different *w* and different concentration ratios *C* are shown in Fig. 5. The optimal lower-bound *E*_L_ always coincides with the bandgap energy *E*_g_. An incident photon can convert only a part of its energy equalling the PV bandgap energy to electricity, while the rest of the photon energy is dissipated as waste heat. Low-energy photons with an energy close to the PV bandgap can be fully utilized by PV cells at high electrical conversion efficiency (relative to high-energy photons). Thus, the optimal lower-bound *E*_L_ always equals the bandgap energy *E*_g_ to maximize the total effective efficiency, considering that thermal energy is normally less valuable than electricity.

The optimal upper-bound *E*_H_ decreases at higher values of *w*. High-energy photons (in the UV spectrum) have a lower electrical conversion efficiency than low-energy photons but also have a high thermal conversion efficiency. Thus, the optimal *E*_H_ decreases as *w* increases, such that more high-energy photons are directed to the thermal absorber as the value of thermal energy increases. The upper limit of *E*_h_ is ~4.4 eV, which is the maximum energy of the incident photons. The optimal bandgap energy for SSPVT collectors increases from 1.05 to 2.05 eV as *w* increases from 0 to 0.8 when *C* = 45,000. Once the values of *w* and *C* of an application are specified, both the optimal PV material (i.e., optimal *E*_g_) and the optimal filter (i.e., optimal *E*_L_ and *E*_H_) can be selected by referring to Fig. 5. It is noteworthy that all energies can be readily converted to wavelengths via the expression *λ*(nm) = 1240/*E*(eV).

### Performance of SSPVT collectors in power-generation applications

The definition and value of *w* depend strongly on the specific application. In this section, the performance of SSPVT collectors in power-generation applications is considered, in which the thermal energy produced by the SSPVT collectors is used downstream to generate secondary electricity via a heat engine. In this case, the total electricity generated by this combined system is equal to that generated by the PV cells in the SSPVT collectors plus that generated by the secondary heat-to-work conversion process.

This section considers SSPVT collectors that are able to produce high-temperature heat, which can be utilized for electricity generation via thermodynamic cycles. The weight coefficient *w* in Eq. (4) can be defined for this particular scenario by considering the thermodynamic value of the heat delivered by the SSPVT collectors. Following a technology agnostic approach, we can define an ideal conversion process from heat to work (electricity) via an ideal (Carnot) heat-engine engine with:

5 $$w = 1 - \frac{{T_{\mathrm{c}}}}{{T_{\mathrm{h}}}}$$

where *T*_h_ is the output (heat delivery) temperature of the SSPVT collector, which is considered to be approximately equal to the temperature of the thermal absorber of the collector, and *T*_c_ is the cold sink temperature of the heat engine (*T*_c_ = 300 K in this study). The term *w* in Eq. (5) presents the highest value of heat relative to that of electricity when the delivered heat is converted to electricity. In this context, the total effective efficiency limits of SSPVT collectors, as expressed in Eq. (4), correspond to the total electrical efficiency of the combined system described above.

**Fig. 6:**
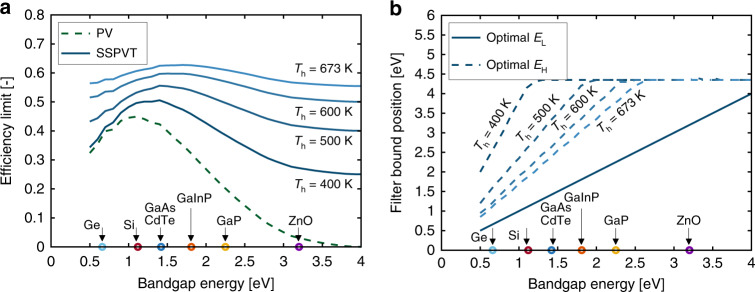
**a** Total effective efficiency limits and **b** optimal lower and upper bounds of spectral-splitting filters for SSPVT power-generation applications (*w* = 1 − *T*_h_/*T*_c_, *C* = 45,000). Output temperatures *T*_h_ = 400, 500, 600 and 673 K correspond to *w* = 0.25, 0.40, 0.50 and 0.55, respectively.

**Fig. 7:**
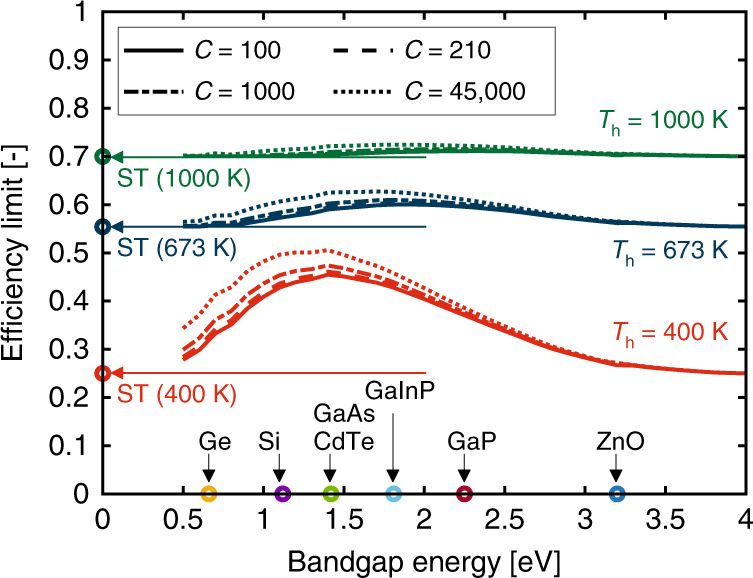
Total effective efficiency limits of SSPVT collectors in power-generation applications for different concentration ratios. Output temperatures *T*_h_ = 400, 500, 600 and 673K correspond to *w* = 0.25, 0.40, 0.50 and 0.55, respectively.

The total SSPVT effective efficiencies at the maximum concentration ratio for different ST output temperatures (*T*_h_ = 400, 500, 600 and 673 K) are shown in Fig. 6a, where 673 K is taken as the maximum limiting temperature of the common high-temperature heat transfer oil Therminol VP-1^39^. At temperatures of 400, 500, 600 and 673 K, we obtain corresponding *w*-values of 0.25, 0.40, 0.50 and 0.55, respectively. From this figure, we observe that integrating SSPVT collectors with ST power generation leads in all cases to significantly higher total electrical efficiencies than standalone PV systems. The total effective efficiency limit of SSPVT collectors increases as the output temperature *T*_h_ increases, driven by the increase in the value of higher-temperature heat. The total effective efficiency limit reaches a peak at 63% at the highest temperature of *T*_h_ = 673 K (*w* = 0.55) but is also sensitive to the bandgap energy of the PV cell material. Seven PV materials with different bandgap energies are marked on the abscissa of Fig. 6a: Ge (0.66 eV), Si (1.12 eV), GaAs (1.42 eV), CdTe (1.43 eV), GaInP (1.81 eV), GaP (2.25 eV) and ZnO (3.20 eV)^40^. The optimal PV materials for the SSPVT collector with *T*_h_ = 400 K (*w* = 0.25) are GaAs and CdTe, which have an efficiency limit of 51%. The selection of the PV material is crucial to SSPVT collectors when *T*_h_ = 400 K (*w* = 0.25), as the efficiency limit is sensitive to the bandgap energy of the cell. The optimal bandgap energy moves to higher values as the output temperature increases. The optimal PV material for SSPVT collectors with *T*_h_ = 673 K (*w* = 0.55) is GaInP. The total effective efficiency limit of SSPVT collectors with *T*_h_ = 673 K varies in the range 55–63%, as the bandgap is varied (corresponding to different PV materials). The selection of the PV material has a slight influence (only 7%) on the total effective efficiency limit of SSPVT collectors when *T*_h_ = 673 K, as indicated in Fig. 7a.

The optimal spectral-splitting filters at four different output temperatures (*T*_h_ = 400, 500, 600 and 673 K) are shown in Fig. 6b. The optimal lower-bound *E*_L_ always equals the bandgap energy of the PV material, whereas the optimal upper-bound *E*_H_ depends not only on the PV material but also on the temperature of the thermal output. Higher-temperature heat is more valuable, as it can be converted to electricity with a higher efficiency. Therefore, the optical filter window narrows as *T*_h_ increases from 400 K to 673 K (as *w* increases from 0.25 to 0.55), indicating that more solar energy is directed to the thermal absorber as less solar energy is directed to the PV cells. For example, the optimal filter for the Si-based SSPVT collector directs only 19% of the incident solar energy to the thermal absorber when *T*_h_ = 400 K, but it directs 62% of the solar energy to the thermal absorber when *T*_h_ = 673 K.

The total effective efficiency limits of SSPVT collectors in power-generation applications for different concentration ratios are shown in Fig. 7. The efficiency limit increases as the concentration ratio *C* increases, owing to the higher PV efficiency at higher concentration ratios. However, although the concentration ratio has an influence on the electrical efficiency and the total effective efficiency, which is noticeable at low temperatures, this decreases at higher temperatures, at which the contribution of the PV electrical output decreases relative to that of the collector’s thermal output. When the temperature reaches *T*_h_ = 1000 K (*w* = 0.70), only 17% of the solar energy is directed to the PV cells and the effect of concentration becomes negligible, as shown in the figure.

Furthermore, from Fig. 7, we can observe that optimum SSPVT collectors have a total efficiency advantage over ST collectors, although this gradually diminishes at progressively higher *T*_h_. The total efficiency limit of SSPVT collectors at lower temperatures (here, 400 K) is close to double that of ideal ST collectors but is only 3–4% higher, in relative terms, when the output temperature is as high as 1000 K.

### Performance of SSPVT collectors with common PV materials

**Fig. 8:**
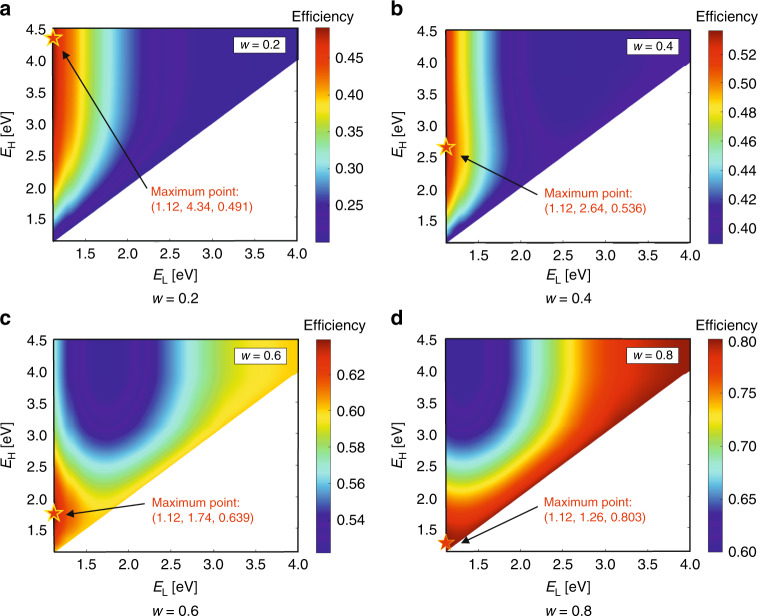
Total effective efficiency for different *E*_L_, *E*_H_ and *w*-values (*C* = 45,000, PV material: Si). The values stated at the maximum points are (*E*_L_, *E*_H_, *η*_tot_).

**Fig. 9:**
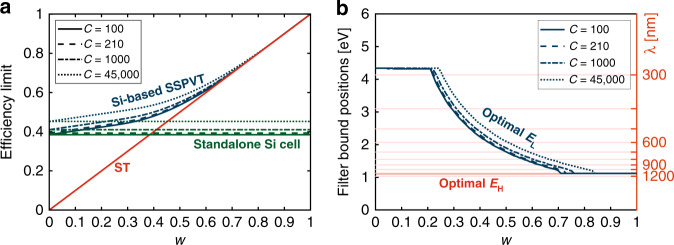
**a** Total effective efficiency limit of Si-based SSPVT collectors, standalone Si cells and ST collectors, and **b** optimal spectral-splitting filter of SSPVT collectors with Si solar cells.

Si is the most common PV material and accounts for over 90% of the current global PV market. The bandgap energy of Si is 1.12 eV (1110 nm). Distributions of the total effective efficiency limits of SSPTV collectors with different spectral-splitting filters for *w* = 0.2, 0.4, 0.6 and 0.8 are shown in Fig. 8. The lower and upper bounds of the spectral-splitting filter significantly affect the total effective efficiency limits of these collectors. The maxima in Fig. 9 are presented in terms of (*E*_L_, *E*_H_, *η*_tot_), which indicate the optimal lower bounds, upper bounds and maximum total effective efficiencies for different *w*. We note that the value of *w* has a significant influence on the total effective efficiency limit and on the optimal filter. The optimal lower bound and upper bound are 1.12 eV and 4.34 eV, respectively, for *w* = 0.2, as shown in Fig. 8a. As the energy limit of solar photons is ~4.4 eV, nearly all incident photons with energy higher than the bandgap energy are directed to the Si solar cell by the optimal filter when *w* = 0.2, corresponding to a scenario where electricity is much more valuable than heat. The optimal upper-bound *E*_H_ decreases from 4.34 to 2.64 eV as *w* increases from 0.2 to 0.4. In this case, more photons with higher energy (in the UV region) are sent to the thermal absorber as the value of thermal energy increases. Interestingly, the optimal lower-bound *E*_L_ is always 1.12 eV for all scenarios, which coincides with the bandgap energy of Si. The optimal upper-bound *E*_H_ approaches the lower-bound *E*_L_ for the case of *w* = 0.8, as shown in Fig. 8d, indicating that nearly all incident solar photons are directed to the thermal absorber when thermal energy has comparable value to that of electricity.

Figure 9a compares the total effective efficiency limits of Si-based SSPVT collectors to those of standalone Si cells and ST collectors for different weights *w* and concentrations *C*. The total effective efficiency of Si-based SSPVT collectors increases significantly at higher *w* or *C*. The electrical efficiency of the standalone Si cell increases from 39% to 45% as *C* increases from 100 to 45,000. The advantage of Si-based SSPVT collectors over standalone ideal Si cell modules becomes larger as *w* increases and the performance of these SSPVT collectors approaches that of the ideal ST collector.

The optimal filters of Si-based SSPVT collectors are shown in Fig. 9b. The left ordinate is in terms of electron energy (eV), whereas the right ordinate is in terms of wavelength (nm). When *w* is <0.2, the optical filter window spans the maximum extent from 1.12 to 4.34 eV (290 to 1110 nm), which coincides with the entire spectral sensitivity (i.e., quantum efficiency) range of Si solar cells. In this case, the maximum extent of the spectrum is directed to the cells, because electricity is more valuable than thermal energy. The filter window for the Si-based collector ‘closes’ at *w* = 0.72 and 0.85 when *C* = 100 and 45,000, respectively, which means that all the solar radiation is directed to the thermal absorber and the PV cell is no longer necessary, corresponding to the scenario in which thermal energy has an equivalent value to electricity. The optimal lower-bound *E*_L_ always equals the bandgap energy of Si 1.12 eV (1110 nm) for all scenarios. The optimal upper-bound *E*_H_ increases as the concentration ratio increases. Figure 9b provides a detailed guidance for selecting an optimal filter for the Si-based SSPVT collector according to the values of *w* and *C* in specific applications.

Other common PV materials include Si, GaAs, CdTe, Cu(In,Ga)(Se,S)_2_ (CIGS) and GaInP^40^. As mentioned earlier, Si cells dominate the current global PV market, followed by CdTe and CIGS^41^. The bandgap energies of the above materials are 1.12 eV (Si), 1.42 eV (GaAs), 1.43 eV (CdTe) and 1.81 eV (GaInP)^40^. The bandgap energy of CIGS can be continuously tuned from ≈1.0 to 2.4 eV by varying the ratios of In/Ga and Se/S^40^. The bandgap of the current record-efficiency CIGS solar cells is ~1.10–1.13 eV^40,42,43^, which is close to the bandgap energy of Si. Thus, the bandgap energy of CIGS is taken as 1.12 eV in this section. Ge (0.66 eV) and GaP (2.25 eV) are also included in the analysis considering their utilization of a very different region of the solar spectrum. In addition, in the context of PVT applications, GaAs has good performance at high temperatures^44^.

**Fig. 10:**
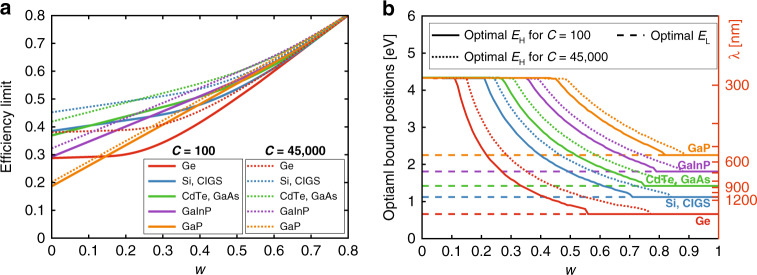
**a** Limits of total effective efficiency and **b** optimal spectral-splitting filters for different common PV cell materials.

The total effective efficiency limits of SSPVT collectors employing the aforementioned PV materials are shown in Fig. 10. As the bandgap of CIGS is close to that of Si, while that of CdTe is close to that of GaAs, Si and CIGS share a single pair of curves in this figure, whereas CdTe and GaAs share another. Figure 10a shows that the limits of the total effective efficiency increase as *w* or *C* increases for all PV materials. The performance of SSPVT collectors with different materials becomes increasingly similar as *w* increases, with the material having a very slight influence on performance for *w* > 0.7. Below this value of *w*, the optimal PV cell material depends strongly on the values of *w* and *C*, so the material needs to be selected carefully, because it determines the ultimate efficiency limit of SSPVT collectors. In general, Si, CIGS, CdTe and GaAs are more suitable for use in SSPVT collectors than in other solar cells, delivering a higher total effective efficiency limit. GaInP gradually catches up as *w* increases. Ge and GaP are largely unsuitable PV cell materials for SSPVT collectors due to their bandgap energies, which are either too low or too high.

The optimal spectral-splitting filters for different PV materials are shown in Fig. 10b. The optimal lower-bound *E*_L_ always equals the bandgap energy of the PV material, which is consistent with earlier observations in this study. Similarly, the upper-bound *E*_H_ decreases as *w* increases, as observed above. The optical filter windows for all PV materials finally cross at certain ‘closing’ points (i.e., the cross points of the curves of optimal *E*_L_ and *E*_H_) as *w* increases. The filter windows for Si/CIGS, CdTe/GaAs and GaInP are close at *w* = 0.85, 0.87 and 0.89, respectively, when *C* = 45,000. The SSPVT collectors operate effectively as ST collectors when *w* exceeds the value at the closing point. The closing point moves rightward as the bandgap energy of the PV cell increases, as shown in Fig. 10b. Figure 10 provides guidance for selecting an optimal PV material and the corresponding optimal spectral-splitting filters that maximize the total effective efficiency limit of SSPVT collectors for different scenarios (i.e., different *w* and *C*).

### Sensitivity analysis of non-ideal factors

In the above sections, we employed an idealized model to determine the total effective efficiency limits of SSPVT collectors. However, in practice, there is a gap between the characteristics of real materials (PV cells, optical filters and thermal absorbers) and those of ideal materials, which will lead to a performance loss. In this section, we consider the impact of non-ideal PV cells, optical filters and thermal absorbers on real SSPVT collector performance. For this purpose, we define two coefficients that describe the non-ideality of real components, one in relation to the PV cells and one to the thermal absorber:

6 $${\mathrm{IC}}_{{\mathrm{PV}}} = \frac{{\eta _{{\mathrm{PV}}\_{\mathrm{act}}}}}{{\eta _{{\mathrm{PV}}\_{\mathrm{idl}}}}}$$

7 $${\mathrm{IC}}_{{\mathrm{th}}} = \frac{{\eta _{{\mathrm{th}}\_{\mathrm{act}}}}}{{\eta _{{\mathrm{th}}\_{\mathrm{idl}}}}}$$

where *η*_PV_idl_ is the efficiency of ideal PV cells with Shockley–Queisser (S-Q) limit efficiencies, *η*_th_idl_ is the efficiency of an ideal thermal absorber with 100% efficiency (see also the justification for this ideal assumption in the present work below Eq. (3)), and *η*_PV_act_ and *η*_th_act_ are the actual efficiencies of real PV cells and thermal absorbers, respectively. The *IC*_PV_ value of Si solar cells reached ≈90% in 2019^45^, whereas the *IC*_th_ value of thermal absorbers in solar towers has also been reported to reach ≈90%^46^.

**Fig. 11:**
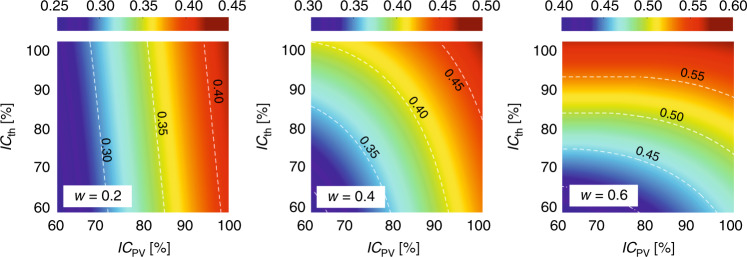
Influence of non-ideal coefficients of the PV cells and thermal absorber, *IC*_PV_ and *IC*_th_, on the SSPVT total effective efficiency limit *η*_tot_ (*C* = 100, PV material: Si).

The influence of the two coefficients above, *IC*_PV_ and *IC*_th_, on the total effective efficiency limit of SSPVT collectors is shown in Fig. 11, with Si selected as the PV material due to its widespread use and a concentration ratio that has been set to *C* = 100, which is readily realized in practice.

We note that the influence of *IC*_PV_ and *IC*_th_ on the SSPVT total effective efficiency limit depends strongly on the value of *w*. Compared to *IC*_th_, *IC*_PV_ has a more significant influence on this efficiency limit for *w* = 0.2, corresponding to scenarios in which electricity is considerably more valuable than heat. Both *IC*_PV_ and *IC*_th_ affect the total effective efficiency limit when *w* = 0.4 and *IC*_th_ becomes the main influencing factor when *w* = 0.6. Therefore, to improve the total effective efficiency limit of real SSPVT systems with non-ideal PV cells and thermal absorbers, the designer should focus on improving *IC*_PV_ when *w* is small (e.g., *w* = 0.2) and on improving *IC*_th_ when *w* is large (e.g., *w* = 0.6).

**Fig. 12:**
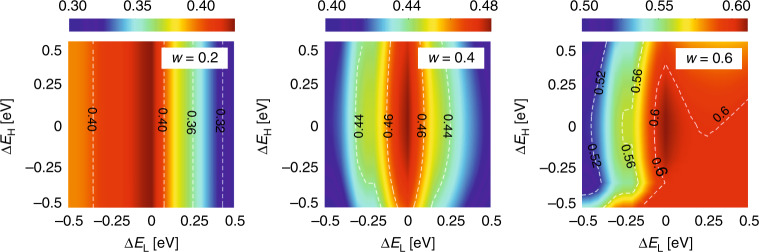
Influence of deviations of *E*_L_ and *E*_H_ from their optimal values on the SSPVT total effective efficiency limit *η*_tot_ (*C* = 100, PV material: Si).

Beyond the PV cells and thermal absorber, the optical filter is another component that can affect the performance of real SSPVT collectors, because, as above, the lower and upper bounds of real filters may deviate from their optimal ideal values. The deviations of these two bounds (i.e., Δ*E*_L_ and Δ*E*_H_) are:

8 $${\Delta}E_{\mathrm{L}} = E_{{\mathrm{L}}\_{\mathrm{act}}} - E_{{\mathrm{L}}\_{\mathrm{opt}}}$$

9 $${\mathrm{{\Delta}}}E_{\mathrm{H}} = E_{{\mathrm{H}}\_{\mathrm{act}}} - E_{{\mathrm{H}}\_{\mathrm{opt}}}$$

where *E*_L_opt_ and *E*_H_opt_ are the lower and upper bounds of the ideal optimal filter, respectively, and *E*_L_act_ and *E*_H_act_ are the real bounds. The influence of Δ*E*_L_ and Δ*E*_H_ (units: eV) on the total effective efficiency limit of SSPVT collectors is shown in Fig. 12.

The deviation Δ*E*_L_ always has a more significant influence on the SSPVT total effective efficiency limit than Δ*E*_H_, which is found to have only a slight influence on this efficiency limit within the range ±0.5 eV. Therefore, it is more important for the designer to ensure that the lower bound of the real optical filter *E*_L_ is close to its optimal value if the ideal optimal cut-off cannot be achieved in practice. The optimal *E*_L_ value always equals the bandgap of the PV cells, e.g., 1.12 eV (1110 nm) for Si cells. Furthermore, a positive deviation of *E*_L_ from its ideal value (i.e., Δ*E*_L_ > 0) leads to a more significant reduction in the total efficiency limit than a negative deviation for *w* = 0.2, whereas a negative deviation (i.e., Δ*E*_L_ < 0) leads to a more significant reduction in the efficiency limit for *w* = 0.6.

Other non-ideal factors that can influence the present results include the PV material bandgap energy, which can shift with temperature if the cells are not sufficiently cooled, and the concentrator optical efficiency. In practice, it may be difficult to cool the PV cells to 300 K at high solar concentrations, with increased PV temperatures usually decreasing the bandgap energy. For example, the bandgap of Si decreases by ≈3% from 1.12 to 1.09 eV as the temperature increases from 300 to 400 K. The effect of this PV bandgap energy shift on the total effective efficiency limits of SSPVT collectors can be seen in Fig. 3, which shows only a slight influence on the efficiency limits that vanishes asymptotically at higher *w*.

## Discussion

We have proposed a framework for predicting the performance of SSPVT collectors, with the aims of identifying the total effective efficiency limits of SSPVT collectors and of providing detailed guidance for selecting optimal PV materials and optimal spectral-splitting filters capable of delivering a combined thermal and electrical performance that reaches the efficiency limits of this technology.

The value of a weighing coefficient *w*, which considers the relative value of thermal energy to that of electricity, has a significant influence on the total effective efficiency limits, the optimal PV cell material and the optimal spectral-splitting filter of ideal SSPVT collectors. The limit of the total effective efficiency increases as either *w* or the concentration ratio, *C*, increase, but is less sensitive to the latter, which suggests that the application is a greater determinant of the ultimate performance of such systems. The total effective efficiency of SSPVT collectors is also particularly sensitive to the bandgap energy of the PV material when *w* is <0.5, corresponding to scenarios in which the electricity is at least twice as valuable as the generated thermal energy. The optimal lower-bound absorption energy of the spectral-splitting filter always equals the bandgap energy of the employed PV material, whereas the upper-bound filter absorption energy decreases as *w* increases. The optical filter window between the two bounds becomes narrower at higher *w*, indicating that more solar energy is directed to the thermal absorber as the thermal output attains a higher value.

SSPVT collectors have an advantage over PV modules that grows monotonically as *w* increases from zero (which is associated with higher thermal-output temperatures, *T*_h_). Specifically, when *w* = 1, SSPVT collectors have ~2.5 times the total efficiency of PV modules. On the other hand, the advantage of ideal SSPVT collectors over ST collectors improves as *w* decreases from unity (which is associated with lower *T*_h_). The total effective efficiency limit of SSPVT collectors in cogeneration applications requiring lower-temperature heat (i.e., ≈100 °C) is approximately double that of ideal ST collectors when considering the relative thermodynamic values of electricity and heat. At intermediate temperatures, SSPVT collectors have the greatest advantage over either of these standalone conventional technologies and systems when both are available for selection and installation. The total effective efficiency limit of SSPVT collectors is over 20% higher than those of both standalone PV modules and ST collectors when *w* is in the range from 0.35 to 0.50 and is up to 30% higher at *w* ≈ 0.4.

The optimal PV cell material for SSPVT collectors depends strongly on the values of *w* and *C*, which in turn are set by the specifics of the application. Suitable PV materials for high-concentration SSPVT collectors are Si and CIGS when *w* < 0.22 or CdTe and GaAs when *w* > 0.22; these same solar cell materials also appear to be the best choice for lower-concentration SSPVT collectors, with GaInP appearing as an additional promising material at higher *w* (above ≈0.4). On the other hand, Ge and GaP emerge from this analysis as less suitable for SSPVT collectors due to their extreme (low/high) bandgap energies. Finally, CIGS cells are considered particularly promising for SSPVT collector applications, owing to their adjustable bandgap energy within the range of ≈1.0–2.4 eV.

The optimal lower and upper bounds of the spectral-splitting filter depend on the PV material, concentration ratio *C* and weighting coefficient *w*.

In summary, detailed maps and other results in this study can assist designers in selecting appropriate PV cell materials and spectral-splitting optical filters, depending on the conditions and application, to achieve optimal overall performance accounting for both energy vectors (i.e., electricity and heat) being provided by these collectors and wider systems.

## Materials and methods

In the model of SSPVT collectors in the main text, the PV electrical efficiency is obtained by a classical PV model. The energy of a photon, corresponding to its wavelength *λ*, can be calculated from:

10 $$E = \frac{{hc}}{{q\lambda }}$$

where *h* is Planck’s constant, *c* is the speed of light, *q* is the elementary charge and *λ* is the photon wavelength.

The number of electron-hole pairs generated in the semiconductor can be calculated from^11^:

11 $$Q_{{\mathrm{PV}}} = {\mathrm{QE}} \cdot {\int}_{\lambda _1}^{\lambda _2} {\frac{{\mathrm{CG}_{\mathrm{s}}(\lambda )}}{{qE}}{\mathrm{d}}\lambda }$$

where QE is the quantum efficiency, *G*_s_(λ) is the incident solar spectral irradiance based on AM1.5, and *λ*_1_ and *λ*_2_ are the lower- and upper-bound wavelengths of the optical filter, corresponding to *E*_H_ and *E*_L_, respectively. The radiative recombination loss of electron-hole pairs can be ignored in an ideal PV cell. Furthermore, the solar concentration ratio *C* is defined as *C* = *A*_a_/*A*_r_, where *A*_a_ is the aperture area of the optical concentrator and *A*_r_ is the receiver area.

The short-circuit current generated by electron-hole pairs can be calculated from:

12 $$I_{{\mathrm{SC}}} = q\cdot Q_{{\mathrm{PV}}}$$

Thus, according to the standard diode equation, the current in a single-junction solar cell under solar illumination is given by^33^:

13 $$I_{{\mathrm{PV}}} = I_{{\mathrm{sc}}} - I_0\left[ {{\mathrm{exp}}\left( {\frac{{qV_{{\mathrm{PV}}}}}{{nk_{\mathrm{b}}T_{{\mathrm{PV}}}}}} \right) - 1} \right]$$

where *V*_PV_ is the applied cell voltage, *k*_b_ is the Boltzmann constant, *T*_PV_ is the temperature of the PV cell and *n* is the ideality factor (=1 in an ideal single *p*–*n* junction solar cell).

In the expression above (i.e., Eq. (13)), *I*_0_ is the dark saturation current, the limit of which is a function of the bandgap energy^33,34^:

14 $$I_0 = I_{00}{\mathrm{exp}}\left( { - \frac{{qE_{\mathrm{g}}}}{{nk_{\mathrm{b}}T_{{\mathrm{PV}}}}}} \right)$$

where *E*_g_ is the bandgap energy of the PV cell in units of eV and where the thermodynamic limit of *I*_00_ is given by Kiess and Rehwald^34^:

15 $$I_{00} = \frac{{2\pi k_{\mathrm{b}}T_{{\mathrm{PV}}}q^3E_{\mathrm{g}}^2}}{{h^3c^2}}$$

Based on the above, the electrical efficiency of the ideal PV cell can be determined from:

16 $$\eta _{{\mathrm{el}}} = \frac{{{\mathrm{max}}\left( {I_{{\mathrm{PV}}} \cdot V_{{\mathrm{PV}}}} \right)}}{{{\int}_0^\infty {\mathrm {CG}_{\mathrm{s}}(\lambda ){\mathrm{d}}\lambda } }}$$

Furthermore, the solar energy absorbed by the thermal absorber is given by:

17 $$E_{{\mathrm{th}}} = {\int}_0^{\lambda _1} {\mathrm{CG}_{\mathrm{s}}(\lambda ){\mathrm{d}}\lambda } + {\int}_{\lambda _2}^\infty {\mathrm{CG}_{\mathrm{s}}(\lambda ){\mathrm{d}}\lambda }$$

Once *E*_th_ is known, the thermal efficiency of the thermal absorber can be determined from:

18 $$\eta _{{\mathrm{th}}} = \frac{{E_{{\mathrm{th}}}}}{{{\int}_0^\infty {CG_{\mathrm{s}}(\lambda ){\mathrm{d}}\lambda } }} - \eta _{{\mathrm{th}},{\mathrm{loss}}}$$

19 $$\eta _{{\mathrm{th}},{\mathrm{loss}}} = \frac{{E_{{\mathrm{loss}}}}}{{{\int}_0^\infty {CG_{\mathrm{s}}(\lambda ){\mathrm{d}}\lambda } }}$$

where *E*_loss_ is the heat loss, including convective and radiative losses from the thermal absorber to the ambient environment, and *η*_th,loss_ is the ratio of the heat loss to the total incident solar energy.

An in-house code was developed to solve the above set of equations. The one-dimensional integrals were solved by a numerical integration method based on Riemann sums^47^. The extreme PV efficiency value (i.e., maximum power point) was identified via a first derivative algorithm, whereas the optimal lower and upper bounds of the optical filter and the optimal PV bandgap were identified via an enumeration algorithm.

**Fig. 13:**
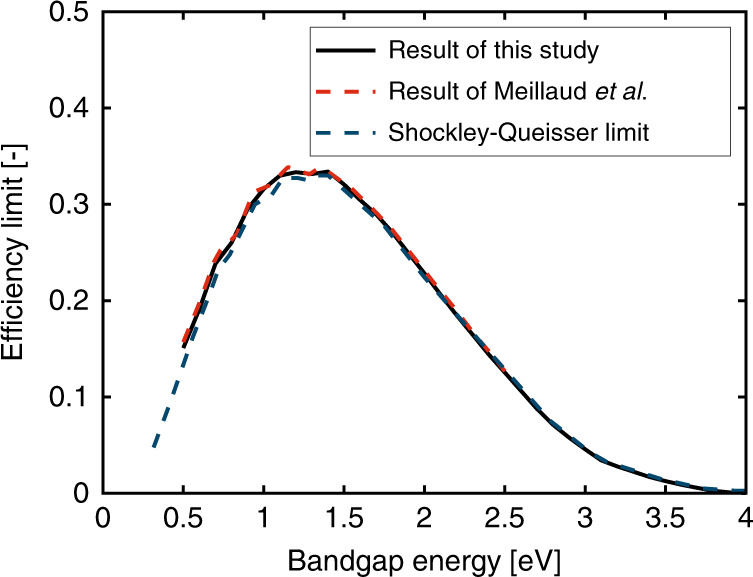
Electrical efficiency limits of single-junction solar cells under the AM1.5 standard predicted by this study, validated against the results from Meillaud et al.^33^, and Shockley and Queisser^11,48^.

The PV model is an important element of the present framework. The electrical efficiency limits of different single-junction solar cells under one sun are calculated using the above model and numerical methodology, and then compared to results from other authoritative publications to validate the model in our study. The well-known S-Q limit has been widely used to estimate the electrical efficiency limits of single-junction solar cells^11,48^. The electrical efficiency limits predicted in our study agree well with the S-Q limit and the recent result from Meillaud et al.^33^, as shown in Fig. 13.
